# Interaction between *TCF7L2* polymorphism and dietary fat intake on high density lipoprotein cholesterol

**DOI:** 10.1371/journal.pone.0188382

**Published:** 2017-11-28

**Authors:** Dhanasekaran Bodhini, Szilvia Gaal, Israa Shatwan, Kandaswamy Ramya, Basma Ellahi, Shelini Surendran, Vasudevan Sudha, Mohan R. Anjana, Viswanathan Mohan, Julie A. Lovegrove, Venkatesan Radha, Karani Santhanakrishnan Vimaleswaran

**Affiliations:** 1 Department of Molecular Genetics, Madras Diabetes Research Foundation, Chennai, India; 2 Hugh Sinclair Unit of Human Nutrition and Institute for Cardiovascular and Metabolic Research (ICMR), Department of Food and Nutritional Sciences, University of Reading, Reading, United Kingdom; 3 Faculty of Health and Social Care, University of Chester, Chester, United Kingdom; 4 Department of Foods, Nutrition and Dietetics Research, Madras Diabetes Research Foundation, Chennai, India; 5 Department of Diabetology, Madras Diabetes Research Foundation, Chennai, India; 6 Dr. Mohan’s Diabetes Specialties Centre, WHO Collaborating Centre for Non-communicable Diseases Prevention and Control, Chennai, India; Tulane University School of Public Health and Tropical Medicine, UNITED STATES

## Abstract

Recent evidence suggests that lifestyle factors influence the association between the Melanocortin 4 receptor *(MC4R)* and Transcription Factor 7-Like 2 *(TCF7L2)* gene variants and cardio-metabolic traits in several populations; however, the available research is limited among the Asian Indian population. Hence, the present study examined whether the association between the *MC4R* single nucleotide polymorphism (SNP) (rs17782313) and two SNPs of the *TCF7L2* gene (rs12255372 and rs7903146) and cardio-metabolic traits is modified by dietary factors and physical activity. This cross sectional study included a random sample of normal glucose tolerant (NGT) (n = 821) and participants with type 2 diabetes (T2D) (n = 861) recruited from the urban part of the Chennai Urban Rural Epidemiology Study (CURES). A validated food frequency questionnaire (FFQ) was used for dietary assessment and self-reported physical activity measures were collected. The threshold for significance was set at P = 0.00023 based on Bonferroni correction for multiple testing [(0.05/210 (3 SNPs x 14 outcomes x 5 lifestyle factors)]. After Bonferroni correction, there was a significant interaction between the *TCF7L2* rs12255372 SNP and fat intake (g/day) (P_interaction_ = 0.0001) on high-density lipoprotein cholesterol (HDL-C), where the ‘T’ allele carriers in the lowest tertile of total fat intake had higher HDL-C (P = 0.008) and those in the highest tertile (P = 0.017) had lower HDL-C compared to the GG homozygotes. In a secondary analysis of SNPs with the subtypes of fat, there was also a significant interaction between the SNP rs12255372 and polyunsaturated fatty acids (PUFA, g/day) (P_interaction_<0.0001) on HDL-C, where the minor allele carriers had higher HDL-C in the lowest PUFA tertile (P = 0.024) and those in the highest PUFA tertile had lower HDL-C (P = 0.028) than GG homozygotes. In addition, a significant interaction was also seen between *TCF7L2* SNP rs12255372 and fibre intake (g/day) on HDL-C (P_interaction_<0.0001). None of the other interactions between the SNPs and lifestyle factors were statistically significant after correction for multiple testing. Our findings indicate that the association between *TCF7L2* SNP rs12255372 and HDL-C may be modified by dietary fat intake in this Asian Indian population.

## Introduction

Genetic variants, unhealthy dietary intake, physical inactivity and their multiple interactions are considered to be contributory factors to the development of obesity and type 2 diabetes (T2D) [1–3]. After China, India has the highest number of people with T2D in the world and according to the Indian Council of Medical Research–INdia DIABetes (ICMR–INDIAB) study, T2D cases have reached 62.4 million and 77.2 million people are pre-diabetic [4]. Furthermore, obesity and T2D are risk factors for non-communicable diseases (NCDs) such as cardiovascular disease (CVD) and it is estimated that India will have the highest rate of CVD mortality in the world [5]. Asian Indians have different biochemical characteristics from other populations from birth, often referred to as the ‘Asian Indian Phenotype’ which consists of increased visceral fat and waist circumference, hyperinsulinemia and insulin resistance [6].

The association between several genes and metabolic diseases has been identified by the candidate gene approach and genome-wide scans; to date, besides the *FTO* (Fat mass and obesity associated) gene, the strongest obesity risk loci known so far [1, 3, 7, 8], two commonly studied candidates for obesity and T2D have been the Melanocortin 4 Receptor (*MC4R)* and Transcription Factor 7-Like 2 (*TCF7L2*) genes. Strong association between the *MC4R* gene and risk of obesity was identified by a genome-wide association (GWAS) study [9] whereas the association between the Transcription Factor 7-Like 2 (*TCF7L2*) gene and risk of T2D was identified by a genome wide linkage study [10]. *MC4R* is expressed in the hypothalamus within the brain and therefore it is suggested that it contributes to body weight regulation by its effect on food intake and energy homeostasis [11]. A strong association was identified between *MC4R* rs17782313 genetic variant and risk of obesity in a European population [12] which was then replicated in other populations [13–15], including Asian Indians [16]. The *TCF7L2* gene is involved in the Wnt signalling pathway where it affects the expression of pro-glucagon and consequently blood glucose regulation [10]. In addition, the effect of pro-glucagon on Glucagon-like peptide 1 (GLP-1) also influences blood glucose regulation with insulin [17]. Decreased insulin secretion and increased glucose production in the liver is suggested to be the result of over expression of the *TCF7L2* gene [18]. A couple of studies [19, 20] have shown strong associations between the two *TCF7L2* single nucleotide polymorphisms (SNPs) (rs7903146, rs12255372) and risk of T2D among Asian Indians living in India, in addition to a meta-analysis [17].

In recent years, several studies have examined whether the association between the genetic variants of *MC4R* and *TCF7L2* genes and cardio-metabolic traits is modified by lifestyle factors such as diet and physical activity in various populations [21–25], however there are no studies to date among Asian Indians [26]. Whilst most studies in European populations found no significant interactions between the *MC4R* SNP rs17782313 and dietary factors on obesity traits [11, 22, 23], a prospective cohort study reported significant interactions between the SNP and fat and protein intake on body mass index (BMI) and risk of T2D [27]. Similarly some studies identified significant interactions between *TCF7L2* SNPs and fibre and fat intake on T2D[28–30]; however there were discrepancies between the studies which could be due to differences in sample size, study design, dietary assessment and genetic heterogeneity. The objectives of this paper were to determine whether the *MC4R* SNP rs17782313 and *TCF7L2* SNPs (rs7903146, rs12255372) were associated with cardio-metabolic traits and whether the association was modified by diet and physical activity in a sample size of up to 1,682 adults from the Chennai Urban Rural Epidemiology Study (CURES).

## Materials and methods

### Study participants

A random, unrelated sample of normal glucose tolerant (NGT) (n = 821) and T2D (n = 861) participants were recruited from the urban part of the cross sectional Chennai Urban Rural Epidemiology Study (CURES) which included 26,001 individuals in total as a representative sample of Chennai city. Full details of the methodology have been explained previously [31]. Briefly 26,001 adult subjects (>20 years of age) were recruited in Phase 1 of CURES using a systematic random sampling method covering the whole Chennai city. This included 1,529 ‘self-reported’ diabetic subjects. In Phase 2, all self-reported or ‘known diabetic’ subjects were invited to our centre for detailed studies of whom 1382 responded (response rate: 90.4%). In Phase 3, every tenth subject from Phase 1, excluding those with self-reported diabetes, underwent an oral glucose tolerance test (OGTT). Subjects who had fasting plasma glucose <5.6 mmol/l (100mg/dl) and 2 hr plasma glucose value 7.8 mmol/l (140mg/dl) were categorized as having NGT [32]. Those who were confirmed by OGTT to have 2 hr plasma glucose value 11.1 mmol/l (200 mg/dl) were classed as ‘newly detected diabetic subjects’ (n = 222).For the present study, the NGT subjects were selected from Phase 3 and subjects with T2D which included ‘known diabetic’ and ‘newly detected diabetic’ subjects were selected from Phase 2 and Phase 3 of the Chennai Urban Rural Epidemiology Study. The Madras Diabetes Research Foundation Institutional Ethics Committee granted the ethical approval and informed consent was obtained from the study participants.

### Anthropometric and biochemical measurements

Weight, height and waist circumference were measured by standard methods. The BMI calculation was based on the body weight (kg) divided by the square of body height (m). Roche Diagnostics (Mannheim) provided the equipments in order to be able to carry out the biochemical analyses on a Hitachi-912 Auto Analyzer (Hitachi, Mannheim, Germany)(8). Fasting plasma glucose, serum cholesterol, serum triglycerides (TG) and high-density lipoprotein cholesterol were measured by glucose oxidase-peroxidase, cholesterol oxidase-phenol-4-amino-antipyrene peroxidase, glycerol phosphatase oxidase-phenol-4-amino-antipyrene peroxidise and polyethylene glycol-pretreated enzyme methods respectively(8). The Friedewald formula was used to estimate low-density lipoprotein cholesterol concentrations [33]. Glycated haemoglobin (HbA1c) and serum insulin were determined by high-performance liquid chromatography (HPLC) on a Variant instrument (Bio-Rad, Hercules, CA, USA) and an enzyme-linked immunosorbent assay (Dako, Glostrup, Denmark) respectively [8].

### Assessment of dietary intake and physical activity

A validated, interviewer administered semi-quantitative food frequency questionnaire (FFQ) [34] consisting of a list of 222 different foods was used in order to evaluate dietary intake for the previous year including macronutrient and total energy intake. Frequencies (per day, week, month, year, never) and portion sizes were estimated by the participants with the help of visual aids of measurement equipments and food sizes. Daily average food and nutrient intake was calculated by the EpiNu database system.

A validated self-report questionnaire was used to measure physical activity [5]. Individuals were divided into the vigorously active group when they both exercised and engaged in demanding work activities whereas within the moderately active group the participants either exercised or carried out heavy physical work. The remainder of the study participants were separated into the sedentary group.

### SNP selection and genotyping

A strong association was identified between *MC4R* rs17782313 genetic variant and risk of obesity in a European population [12] which was then replicated in other populations (13–15), including Asian Indians [16]. Strong associations have also been found between *TCF7L2* SNPs (rs12255372 and rs7903146) and risk of T2D in a meta-analysis [17] in the Japanese population [35] as well as in Asian Indians [19, 20]. Although the *TCF7L2* SNPs (rs12255372 and rs7903146) have showed significant linkage disequilibrium (r^2^ = 0.746), the rs7903146 variant has been shown to have the strongest effect in Caucasian populations [36, 37]. Based on the previous studies, the above mentioned three SNPs were selected for the present study.

Phenol-chloroform method was used to extract DNA from whole blood. The methodology for genotyping *TCF7L2* rs12255372 (G/T) and rs7903146 (C/T) SNPs has been previously published [20]. Direct sequencing by an ABI 310 genetic analyzer (Applied Biosystems, Foster City, CA) helped to confirm the efficiency of the genotyping which was in 99% concordance based on random duplicates of 20% of the samples [20].

*MC4R* rs17782313 (T/C) SNP: PCR volume (10 μl) consisted of 1X reaction buffer, 200 mmol of dNTP, 1.5 mmol of MgCl_2_, 1 U taq DNA polymerase and 100 ng of genomic DNA. The concentrations for primers included 15 pmol of common primer, 15 pmol of allele 1 primer and 1 pmol of allele 2 primer, equal to a 15:1 ratio of short primers to long primer. Cycles for PCR were carried out at 96°C for 12 min, then 35 cycles at 94°C for 30 sec, followed by 30 sec at 57°C, 30 sec at 72°C and finally for 10 min at 72°C. Electrophoresis was carried out with a 3% agarose gel.

Inner primers

F: AAGTTTAAAGCAGGAGAGATTGTATACC (C allele 222bp)

R: GCTTTTCTTGTCATTTCCAGCA (T allele 149bp)

Outer primer

F: TTACTGATTTTAAGGGCATAAGCAA

R: TATCATGCTGAGACAGGTTCATAAA (321bp)

### Statistical analyses

Statistical analyses were carried out by using SPSS software (version 21). BMI ≥ 25 kg/m^2^ was categorised as obese and BMI < 25 kg/m^2^ as non-obese. Descriptive statistics for continuous variables are shown as means and standard deviation (SD). Fasting serum insulin and triglyceride values were log transformed to obtain normal distribution. Genotype frequencies between cases and controls were compared by Chi Square test. The difference in the means of continuous variables between the genotypes was analysed by independent sample t test. Based on an additive model of analysis, dominant models were used for all 3 single nucleotide polymorphisms (SNPs) where the common homozygous allele was compared to the combined heterozygous and rare homozygous alleles due to the low allele frequency of rare homozygotes. Association analyses between SNPs and continuous and categorical variables were carried out by linear and logistic regression models, respectively, adjusting for age, gender with the addition of BMI, when T2D was the outcome, and, adjusting for T2D, when obesity was the outcome. Linear and logistic regression models were also used for interaction analyses between SNPs and dietary factors (continuous variables) / physical activity (categorical variable) on continuous and categorical outcomes respectively, where the interaction terms were included into the models and were adjusted for age, gender, BMI, T2D and total energy intake when appropriate. Bonferroni correction for multiple testing was calculated by multiplying 3 SNPs with 14 outcomes (T2D, obesity, BMI, waist circumference, fasting blood glucose, HbA1c, fasting insulin, systolic and diastolic blood pressure, HDL, LDL, VLDL, TG and total cholesterol) and 4 dietary factors (carbohydrate, protein, fat, fibre) and physical activity level. The P value of 0.05 was then divided by 210 (3 SNPs x 14 outcomes x 5 lifestyle factors) which set the significant p value for all results at P = 0.00023.

As a secondary analysis, given the significant SNP-fat intake interaction, individuals were grouped into tertiles based on the fatty acid subtypes [monounsaturated (MUFA) and polyunsaturated (PUFA) fatty acids] for testing the interaction between SNPs and these fatty acid subtypes on lipids. In addition, PUFA was further stratified into ALA (alpha linolenic acid) and LA (linoleic acid) for the interaction analysis.

Given that there are no previously reported effect sizes for the gene-diet interaction, we were unable to perform a prospective power calculation. However, based on the most significant interaction observed in the present study, we performed retrospective power calculations using QUANTO software, Version 1.2.4 (May 2009). We performed power calculations in the form of least detectable effects based on the assumption of significance levels and powers of 5 and 80%, respectively. At 80% power, the minimum detectable effects ranged from beta 0.02 mg/dl (HDL-C) for a SNP with MAF of 5% to beta 1.0 mg/dl for a SNP with MAF 50% in the case-control analysis. For the *TCF7L2* SNP–fat intake interaction on HDL-C (most significant interaction), the beta was 0.067, which is within the range of effect sizes for which the power was calculated.

## Results

### Phenotypic associations

Based on the clinical and biochemical characteristics of the individuals from the CURES study as illustrated in **Table 1,** individuals with T2D were older (P<0.0001), had higher BMI (P<0.0001), waist circumference (P<0.0001), fasting plasma glucose (P<0.0001), HbA1c (P<0.0001), fasting plasma insulin (P<0.0001), systolic and diastolic blood pressure (P<0.0001), low density and very low density lipoproteins (P<0.0001), total cholesterol (P<0.0001), TG (P<0.0001) and lower high density lipoprotein (P = 0.001) than NGT individuals.

**Table 1:** Anthropometric and biochemical characteristics of T2D and NGT participants.

	NGT	T2D	P value*
**N**	821	861	
**(men/women)**	(345/476)	(398/463)	0.08
**Age (yrs)**	41.31±11.73	50.57±10.49	<0.0001
**BMI (kg/m**^**2**^**)**	23.66±4.69	25.34±4.30	<0.0001
**WC (cm)**	83.55±11.66	90.60±9.84	<0.0001
**FPG (mg/dl)**	84.82±8.36	161.73±69.13	<0.0001
**HbA1c (%)**	5.58±0.48	8.78±2.36	<0.0001
**INS (μIU/ml)**	8.41±5.81	11.78±7.69	<0.0001
**Log INS (μIU/ml)**	6.85±1.87	9.68±1.90	<0.0001
**Systolic BP (mmHg)**	117.72±18.05	129.16±21.65	<0.0001
**Diastolic BP (mmHg)**	74.06±11.32	76.76±11.85	<0.0001
**HDL (mg/dl)**	43.36±9.91	41.71±9.50	0.001
**LDL (mg/dl)**	113.13±30.55	124.49±35.38	<0.0001
**VLDL (mg/dl)**	23.95±14.30	35.96±27.37	<0.0001
**TC (mg/dl)**	180.02±36.02	199.51±42.40	<0.0001
**TG (mg/dl)**	120.18±71.46	180.31±137.12	<0.0001
**Log TG (mg/dl)**	105.68±1.62	152.75±1.71	<0.0001
**Total energy (Kcal/day)**	2622.43±702.77	2468±893.75	<0.0001
**Total Carbohydrate (g)**	419.69±112.33	399.96±147.98	0.002
**Fat (g)**	68.57±24.29	64.25±27.31	0.001
**Total PUFA (g)**	18.22±9.09	18.75±10.04	0.25
**Total MUFA (g)**	20.51±7.77	18.79±8.37	<0.0001
**Protein (g)**	73.82±20.97	69.93±24.56	0.001
**Dietary fibre (g)**	31.41±9.83	31.66±12.19	0.64

Data presented as Mean±SD.

*P values are showing the differences in mean values between NGT and T2D participants.

*Abbreviations*: NGT, normal glucose tolerance; BMI, Body mass index; WC, waist circumference; HbA1C, glycated haemoglobin; FPG, Fasting plasma glucose; INS, Fasting plasma insulin; HDL, high density lipoprotein; LDL, low density lipoprotein; VLDL, very low density lipoprotein; TC, Total Cholesterol; TG, triglycerides.

### Genetic associations

The minor allele (‘T’) of SNPs (rs12255372 and rs7903146) of *TCF7L2* gene showed significant susceptibility to T2D; however, in the present study after correction for multiple testing only the association between SNP rs7903146 and T2D remained statistically significant (P = 0.0001) (**S1 Table**).After Bonferroni correction, statistically significant association between the ‘C’ allele of the*MC4R* SNP rs17782313 and T2D was also observed (P = 0.00022) (**S2 Table**). None of the associations between the three SNPs and continuous variables remained significant after correction for multiple testing (P>0.00023) (**S1 and S2 Tables**).

### *TCF7L2 –*dietary fat intake interactions on HDL-C

Individuals were grouped into tertiles based on their fat and subtypes of fat intake (g/day). The means for fat intake in the 1^st^ tertile: 41 g/d; 2^nd^ tertile: 62 g/d; 3^rd^ tertile: 95 g/d. Means for PUFA intake in the 1^st^ tertile: 9g/d; 2^nd^ tertile: 17 g/d; 3^rd^ tertile: 29 g/d. Means for MUFA intake in the 1^st^ tertile: 12 g/d; 2^nd^ tertile: 18 g/d; 3^rd^ tertile: 29 g/d. Means for ALA intake in the 1^st^ tertile: 0.38 g/d; 2^nd^ tertile: 0.58 g/d; 3^rd^ tertile: 0.89 g/d. Means for LA intake in the 1^st^ tertile: 8 g/d; 2^nd^ tertile: 17 g/d; 3^rd^ tertile: 29 g/d.

**Fig 1:**
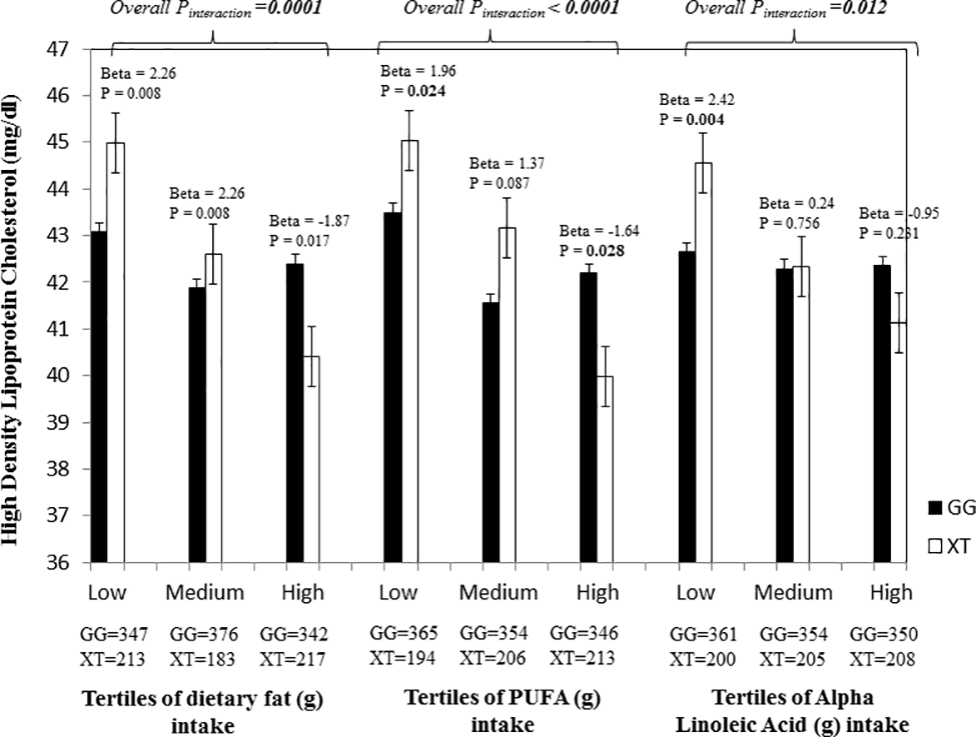
Interaction of the *TCF7L2* gene polymorphism (rs12255372) with fat (g) intake, PUFA intake and Alpha Linolenic Acid (g) intake on HDL-C. Individuals carrying the ‘XT’ genotype had 2.26 mg/dl higher HDL-C in the lowest fat tertile (P = 0.008), while those in the highest tertile had 1.87 mg/dl lower HDL-C (P = 0.017) than those who carry the ‘GG’ allele. Carriers of the ‘XT’ genotype had 1.96 mg/dl higher HDL-C in the 1^st^ tertile of PUFA intake (g) (P = 0.024), while those in the 3^rd^ tertile had 1.64 mg/dl lower HDL-C in comparison to the carriers of the ‘GG’ genotype (P = 0.028). In the 1^st^ tertile of Alpha Linolenic acid intake (g), individuals with the ‘XT’ genotype had 2.42 mg/dl higher HDL-C than the ‘GG’ homozygotes (P = 0.004).

The interaction between the *TCF7L2* SNP rs12255372 and fat intake (g/day) on HDL-C was statistically significant after correction for multiple testing (P_interaction_ = 0.0001). The ‘T’ allele carriers of the *TCF7L2* SNP rs12255372 had 2.26 mg/dl higher HDL-C level in the lowest tertile of fat intake (mean: 41 g/day) than the ‘GG’ homozygotes (P = 0.008) and in the highest tertile of fat intake (mean: 95 g/day), HDL-C was 1.87 mg/dl lower in the risk ‘T’ allele carriers in comparison to the ‘GG’ homozygotes (p = 0.017) (**Fig 1**).

Stratification to fat subgroups showed significant interactions between the SNP rs12255372 and PUFA (g/day) on HDL-C (P_interaction_<0.0001), where the ‘T’ allele carriers had 1.96 mg/dl higher HDL-C (P = 0.024) in the low PUFA tertile (mean: 9 g/day) in comparison to the ‘GG’ homozygotes and in the 3^rd^ tertile (mean: 29 g/day), the HDL-C level of the ‘T’ allele carriers was 1.64 mg/dl lower than the ‘GG’ homozygotes (P = 0.028) (**Fig 1**). A similar interaction was also found between the SNP rs12255372 and MUFA (g/day) on HDL-C (P_interaction_ = 0.0003), where the ‘T’ allele carriers had 1.77 (mg/dl) higher HDL-C in the lowest MUFA tertile (mean: 12 g/day) (P = 0.03) and had 1.61 (mg/dl) higher HDL-C in the 2nd tertile (mean: 18 g/day) (P = 0.045) than the ‘GG’ carriers, however in the highest MUFA tertile (mean: 29 g/day) the ‘T’ allele carriers had 1.59 (mg/dl) decreased HDL-C (P = 0.041) than individuals with the ‘GG’ genotype.

PUFA was further stratified to linoleic acid (LA) and alpha linolenic acid (ALA) to investigate whether omega-3 and omega-6 fatty acids modified the association between the *TCF7L2* SNP rs12255372 and HDL-C. Significant interaction was found between the SNP and ALA on HDL-C (P_interaction_ = 0.012), where the ‘T’ allele carriers had 2.42 (mg/dl) higher HDL-C than the ‘GG’ homozygotes (P = 0.004) in the lowest tertile (mean: 0.38 g/day) (**Fig 1**).A similar interaction was also found between the SNP rs12255372 and LA (g/day) on HDL-C (P_interaction_<0.0001). **S1 Table** shows the interactions of *TCF7L2* SNP rs12255372 with fat, PUFA and ALA intakes on HDL-C under an additive and dominant model.

### Additional gene-diet interactions

In addition to the main significant findings, there was a significant interaction between *TCF7L2* SNP rs12255372 and fibre intake (g/day) on HDL-C (P_interaction_< 0.0001), where in the lowest tertile (mean: 20 g/day) individuals carrying the ‘T’ allele had 1.95 (mg/dl) higher HDL-C (P = 0.02), in the 2^nd^ tertile (mean: 30 g/day), the ‘T’ allele carriers had 2.39 (mg/dl) higher HDL-C (P = 0.003) and in the highest tertile (mean: 44 g/day), the HDL-C level of the ‘T’ allele carriers was 2.37 mg/dl lower in comparison to the ‘GG’ homozygotes (P = 0.002). There were several other interactions which did not reach statistical significance after correction for multiple testing and these interactions are shown in the **Table 2** and **S2 and S3 Tables**).

**Table 2:** Interactions of *TCF7L2* SNPs rs12255372 and rs7903146 with carbohydrate, fibre and protein intake on HDL-C, fasting blood glucose and diastolic blood pressure.

**rs12255372**	SNP*Carbohydrate (g)	SNP*Fibre (g)	SNP*Protein (g)	SNP*Fat (g)
β ± SE** (P for interaction on FPG)	0.039 ± 0.019 (0.041)	0.437 ± 0.222 (0.049)	0.132 ± 0.108 (0.222)	0.119 ± 0.095 (0.210)
β ± SE** (P for interaction on HDL-C)	0.009 ± 0.003 (0.007)	0.168 ± 0.041 (<0.0001)	0.072 ± 0.020 (0.0003)	0.067 ± 0.017 (0.00017)
β ± SE** (P for interaction on DBP)	-0.003 ± 0.004 (0.551)	-0.107 ± 0.050 (0.033)	-0.028 ± 0.024 (0.251)	-0.032 ± 0.021 (0.130)
**rs7903146**	SNP*Carbohydrate (g)	SNP*Fibre (g)	SNP*Protein (g)	SNP*Fat (g)
β ± SE** (P^¥^ for interaction on T2D)	0.002 ± 0.001 (0.011)	0.014 ± 0.010 (0.180)	0.011 ± 0.005 (0.024)	0.008 ± 0.004 (0.071)
β ± SE** (P for interaction on HDL-C)	0.006 ± 0.003 (0.057)	0.128 ± 0.040 (0.002)	0.050 ± 0.020 (0.010)	0.052 ± 0.017 (0.003)

*Interaction term

**P values are adjusted for age, gender, BMI, T2D and Total energy intake.

^¥^ P values are adjusted for age, gender, BMI and Total energy intake.

*Abbreviations*: SNP, Single nucleotide polymorphism; HDL-C, High density lipoprotein cholesterol; FPG, Fasting plasma glucose; DBP, Diastolic blood pressure.

### Gene-physical activity interactions on cardio-metabolic traits

No statistically significant interactions were observed after correction for multiple testing between the three SNPs and physical activity on obesity- and T2D- related traits (P_interaction_>0.0002). Interactions observed between *TCF7L2* SNP rs7903146 and physical activity on VLDL (P_interaction_ = 0.012) and TG (P_interaction_ = 0.014), where the risk ‘T’ allele carriers had higher TG and VLDL levels, did not reach statistical significance after correction for multiple testing **(S4 Table).**

## Discussion

This is the first study to investigate interactions between *TCF7L2* and *MC4R* SNPs and lifestyle factors on cardio-metabolic traits among Asian Indians. The main findings suggest that total fat and PUFA intakes may modify the association between the *TCF7L2* SNP rs12255372 and HDL-C. In the lowest total fat and PUFA tertiles, participants carrying the ‘XT’ genotype (GT+TT) had significantly higher HDL-C, whereas in the highest tertile participants carrying the ‘XT’ genotype had lower HDL-C, as compared to those with the GG genotype. This finding is of public health significance given that Asian Indians tend to have low HDL-C which puts them at markedly increased risk for CVD [38, 39].

The fat intake consists of invisible, hidden and visible fat (vegetable oils, ghee, butter) in India where in the urban areas the minimum average daily intake of visible fat is approximately 22–45 g/day [40]. The recommended omega 3 PUFA intake is 0.1 gram/day, however the median of combined intake of LA and ALA is 13 g/day and the recommended ratio between LA and ALA is 5:1 to 10:1 [40]. In comparison, the results of our study suggest that total fat intake below 53 g/day, PUFA intake below 13 g/day, and ALA intake below 0.49 g/day may help maintain high levels of HDL-C in the ‘T’ allele carriers of the *TCF7L2* SNP rs12255372. The findings that MUFA intake above 22 g/day reduces HDL-C and only ALA intake below 0.5 g/day maintains high HDL-C level in risk carriers are unexpected results; however, the effect of polygenic traits cannot be ruled out.

HDL-C is generally considered to be protective against CVD due to its role in the reverse cholesterol transport; however, the recent Mendelian randomization (MR) studies [41, 42] have failed to show a causal effect of low HDL-C on cardiovascular disease risk. But, none of these MR studies have taken dietary factors into account. Furthermore, fatty acids have shown to have different modulating effect on HDL-C for which the mechanism is not fully understood [43]. On the other hand, it is argued that the total fat intake is more influential on postprandial lipoprotein abnormalities, which is a characteristic of T2D, than the type of fatty acids [44]. However, studies have also shown that different fatty acids can have differential effects on postprandial TG [45–47]. In our study, we found that those in the lowest fat intake subgroups had higher HDL-C while those in the highest tertile groups had lower HDL-C among the risk allele carriers. A previous intervention study in the US population [30] also found significant interaction between SNP rs12255372 and fat intake where positive changes in body composition were observed in the ‘T’ risk allele carriers only on the low-fat diet (20% from total energy). Other studies only reported significant interactions between SNP rs7903146 and high saturated fat intake on increased metabolic syndrome risk [29] and high (n-6) PUFA intake (≥6.62% of energy intake) on increased VLDL and TG [24] among the risk ‘T’ allele carriers.

High omega 6 to omega 3 ratio has been shown to increase HDL in mice [43], whereas the results from this study in humans contradict those findings. It has been shown in cell and animal studies that high omega 6 PUFA intake is pro-inflammatory leading to an increased risk for CVD and diabetes [48] and in the present study reduced HDL was observed in the minor allele carriers in response to high intake of omega 6 PUFA (>21.6 g/d). According to our findings, a low omega 6 (<12.8 g/d) and a low ALA intake (<0.5 g/d) may help maintain HDL above 44 (mg/dl) in the risk allele carriers.

Though there was no significant difference in mean HDL levels in the different tertiles of total fat and PUFA intakes in the study participants (data not shown), in the presence of *TCF7L2* rs12255372 genotype, a clear interaction between the genotype and fat/ PUFA intake on HDL-C was observed. While the carriers of ‘XT’ genotype had increased HDL-C in the presence of a low fat/ low PUFA diet, there was a decrease in HDL-C levels in the carriers of ‘XT’ genotype in the presence of a high fat / high PUFA diet. It is to be noted that among those who carry the GG genotype, irrespective of the dietary fat/PUFA intake, there is no effect on HDL-C levels.

Another interesting interaction was the one between *TCF7L2* SNP rs12255372 and fibre intake on HDL-C. The results suggest that low and medium fibre intake (means: 20, 30 g/day), respectively, increase HDL-C whereas high fibre intake (mean: 44 g/day) may reduce HDL-C among risk allele carriers of the *TCF7L2* SNP rs12255372. The average fibre intake in India is 30–40 g/day [40] which is consistent with the mean intake in the medium and high tertiles and also higher compared to the mean fibre intake in the UK (~18g/day) and the US (~16g/day). A previous study investigated interaction between SNP rs7903146 and fibre intake on T2D risk [28],where the minor allele carriers had increased T2D risk with higher fibre intake (mean intake: 13.1 ± 2.2 g/4,184 kJ). High fibre intake is generally recommended to improve glycemic control in T2D individuals [49] and to reduce total and LDL-C in order to reduce CVD risk. A meta-analysis of data from clinical studies (n = 2,990) [50] indicated that high fibre diets (20–30 g/day) reduced HDL-C. Similarly, another meta-analysis of data from 24 clinical studies also suggested that medium and high carbohydrate, high fibre (≥20 g/day) diets also decreased HDL by 4% [51].However, it was argued, that the decrease in LDL-C and TG values would reduce CVD risk by 16.4% which would outweigh the increased risk of CVD by 11.9% due to decreased HDL [51]. But it is of note that these meta-analyses did not consider the genetic component and, furthermore, we also observed gene-diet interaction on other lipid outcomes such as VLDL and TG, which did not remain significant after correction for multiple testing.

The risk of coronary artery disease is increased in individuals with circulating HDL-C concentration of <40 mg/dl [52]. In our study, within the high fibre intake tertile (≥35g/day), the mean HDL-C level of the minor risk allele carriers was 39.46 mg/dL in comparison to 42.27 mg/dl of the ‘GG’ carriers of the *TCF7L2* SNP rs12255372. The results of our study suggest that high fibre intake (35–102 g/day) may help maintain HDL-C level above 40 mg/dl whereas below 35 g/day may lower HDL-C in the ‘GG’ genotype carriers of SNP rs12255372. Mechanisms on how low/high fibre intake decreases/increases HDL-C are not well understood and more research is needed to clarify the effects of dietary fibre on HDL-C metabolism [52, 53].

No studies, to date, have investigated interactions between the *TCF7L2* SNPs (rs12255372 and rs7903146) and *MC4R* SNP rs17782313 and physical activity on cardio-metabolic traits in Asian Indians. Despite the fact that the majority of people are physically inactive in India [54], no significant interactions were found after correction for multiple testing between the three polymorphisms and physical activity on cardio-metabolic traits, which could be due to a small sample size and measurement bias associated with self-reported physical activity questionnaire. However, our finding is in support of the previous study in a Spanish population [21] with a much larger sample size (n = 7,052) which also did not find a significant interaction between *MC4R* SNP rs17782313 and physical activity on obesity traits. Though the inclusion of a representative sample of Chennai for analysis and the use of a comprehensive, validated, interviewer administered semi-quantitative FFQ for dietary assessment could be considered the strengths of this study, there are some underlying limitations. The data used to calculate the measures of dietary intake and physical activity came from self-report and hence, measurement bias associated with self-reported questionnaire cannot be ruled out. Given that obesity and diabetes are multifactorial traits, several genetic and lifestyle factors are likely to contribute to the disease. While 97 loci have been shown to be associated with body weight [55], the present study examined only three common variants, given their consistent associations with obesity and diabetes, respectively, in Europeans and Asian Indians. The cross sectional study design gives only a snapshot of the prevalence and cause—effect cannot be established due to lack of follow up which is another limitation of this study. However, several outcomes and risk factors were assessed and there was no loss to follow up.

In conclusion, this study has found significant interactions between the *TCF7L2* SNP rs12255372 and dietary factors on HDL-C in this Asian Indian population. The results of this study indicate that high total fat and PUFA intakes may be associated with lower HDL-C whereas low intake is associated with higher HDL among the risk allele carriers. More research is required to better understand the interactions between the *TCF7L2* gene variant and lifestyle factors on cardio-metabolic traits. Exact mechanisms identifying the effect of different fatty acids on HDL-C and whether/how high fat- and PUFA- intake may reduce HDL-C should also be established before public health recommendations and personalised nutrition advice can be developed for this Asian Indian population in order to reduce the burden of cardiometabolic diseases.

## Supporting information

S1 Table Interactions of *TCF7L2* SNPs rs12255372 with fat, PUFA and ALA intakes on HDL-C under an additive and a dominant model of analysis. (DOCX)

S2 Table Associations between *TCF7L2* SNPs rs12255372, rs7903146 and obesity, T2D and related traits. (DOCX)

S3 Table Association and interaction between *MC4R* SNP rs17782313 and obesity, T2D and related traits. (DOCX)

S4 Table Interactions between *TCF7L2* SNP rs7903146 and physical activity on VLDL and TG. (DOCX)

## Abbreviations

*MC4R*: Melanocortin 4 receptor
*TCF7L2*: Transcription factor 7 like 2
CURES: Chennai Urban Rural Epidemiological Study
NGT: Normal glucose tolerance
T2D: Type 2 diabetes
