# Supplementary material for: Interaction between TCF7L2 polymorphism and dietary fat intake on high density lipoprotein cholesterol
Source: PLoS One. 2017 Nov 28;12(11):e0188382. doi: 10.1371/journal.pone.0188382 (PMC5705148; doi:10.1371/journal.pone.0188382)
Supplement: S1 Table — (DOCX) [file pone.0188382.s001.docx]

**Online Supporting Material**

**S1 Table:** Interactions of *TCF7L2* SNPs rs12255372 with fat, PUFA and ALA intakes on HDL-C under an additive and a dominant model of analysis

| **Tertiles** | **Genotype** | **N** | **HDL-C** | | **Additive**  **Model** | | | | **Dominant Model** |
| --- | --- | --- | --- | --- | --- | --- | --- | --- | --- |
|  |  |  | **Mean** | **SE** | **P_association_ GGvsGT** | **P_association_ GGvsTT** | **P_association_ GTvsTT** | **Overall P_interaction_** | **P_association_**  **GGvsXT** |
| Fat Tertile 1 | GG | 347 | 42.946 | 0.520 | 0.007 | 0.356 | 0.580 | **0.001** | 0.008 |
|  | GT | 173 | 45.383 | 0.737 |  |  |  |  |  |
|  | TT | 40 | 44.441 | 1.533 |  |  |  |  |  |
|  | XT (GT+TT) | 210 | 45.200 | 0.664 |  |  |  |  |  |
| Fat Tertile 2 | GG | 376 | 41.667 | 0.442 | 0.080 | 0.683 | 0.700 |  | 0.086 |
|  | GT | 158 | 43.099 | 0.684 |  |  |  |  |  |
|  | TT | 25 | 42.387 | 1.709 |  |  |  |  |  |
|  | XT (GT+TT) | 183 | 43.000 | 0.634 |  |  |  |  |  |
| Fat Tertile 3 | GG | 342 | 42.349 | 0.487 | 0.016 | 0.583 | 0.687 |  | 0.017 |
|  | GT | 196 | 40.402 | 0.643 |  |  |  |  |  |
|  | TT | 21 | 41.236 | 1.965 |  |  |  |  |  |
|  | XT (GT+TT) | 217 | 40.480 | 0.611 |  |  |  |  |  |
|  |  |  |  |  |  |  |  |  |  |
| PUFA Tertile 1 | GG | 365 | 43.345 | 0.508 | 0.021 | 0.606 | 0.641 | **0.011** | 0.024 |
|  | GT | 171 | 45.429 | 0.744 |  |  |  |  |  |
|  | TT | 23 | 44.424 | 2.025 |  |  |  |  |  |
|  | XT (GT+TT) | 194 | 45.310 | 0.698 |  |  |  |  |  |
| PUFA Tertile 2 | GG | 354 | 41.646 | 0.483 | 0.083 | 0.584 | 0.710 |  | 0.087 |
|  | GT | 169 | 43.132 | 0.701 |  |  |  |  |  |
|  | TT | 37 | 42.514 | 1.506 |  |  |  |  |  |
|  | XT (GT+TT) | 206 | 43.020 | 0.635 |  |  |  |  |  |
| PUFA Tertile 3 | GG | 346 | 41.983 | 0.458 | 0.025 | 0.603 | 0.640 |  | 0.028 |
|  | GT | 187 | 40.240 | 0.624 |  |  |  |  |  |
|  | TT | 26 | 41.078 | 1.676 |  |  |  |  |  |
|  | XT (GT+TT) | 213 | 40.340 | 0.585 |  |  |  |  |  |
|  |  |  |  |  |  |  |  |  |  |
| ALA Tertile 1 | GG | 365 | 43.371 | 0.508 | 0.022 | 0.380 | 0.898 | **0.004** | 0.019 |
|  | GT | 170 | 45.444 | 0.746 |  |  |  |  |  |
|  | TT | 24 | 45.172 | 1.984 |  |  |  |  |  |
|  | XT (GT+TT) | 194 | 45.410 | 0.690 |  |  |  |  |  |
| ALA Tertile 2 | GG | 353 | 41.594 | 0.483 | 0.067 | 0.784 | 0.504 |  | 0.087 |
|  | GT | 171 | 43.158 | .696 |  |  |  |  |  |
|  | TT | 36 | 42.034 | 1.528 |  |  |  |  |  |
|  | XT (GT+TT) | 207 | 42.960 | 0.630 |  |  |  |  |  |
| ALA Tertile 3 | GG | 347 | 41.988 | 0.458 | 0.024 | 0.598 | 0.636 |  | 0.026 |
|  | GT | 186 | 40.223 | 0.626 |  |  |  |  |  |
|  | TT | 26 | 41.071 | 1.676 |  |  |  |  |  |
|  | XT (GT+TT) | 212 | 40.320 | 0.580 |  |  |  |  |  |

PUFA, Polyunsaturated fatty acid; ALA, Alpha linoleic acid; HSL-C, H High Density Lipoprotein Cholesterol
