# Supplementary material for: Interaction between TCF7L2 polymorphism and dietary fat intake on high density lipoprotein cholesterol
Source: PLoS One. 2017 Nov 28;12(11):e0188382. doi: 10.1371/journal.pone.0188382 (PMC5705148; doi:10.1371/journal.pone.0188382)
Supplement: S2 Table — (DOCX) [file pone.0188382.s002.docx]

**S2 Table: Associations between *TCF7L2* SNPs rs12255372, rs7903146 and obesity, T2D and related traits.**

|  | **rs12255372**  **OR (95% CI)**  **P value** | **rs7903146**  **OR (95% CI)**  **P value** |
| --- | --- | --- |
| Obesity | 0.92 (0.75-1.13)  0.44^a^ | 0.80 (0.66-0.98) **0.03^a^** |
| T2D | 1.39 (1.11-1.73)  **0.004**^b^ | 1.53 (1.23-1.89) **0.0001^b^** |

^a^P value is adjusted for age, gender and T2D

^b^P value is adjusted for age, gender and BMI

|  | **rs12255372** | | | **rs7903146** | | |
| --- | --- | --- | --- | --- | --- | --- |
|  | **Genotype: GG**  **(n=1067)** | **Genotype: XT (GT + TT) (n=615)** | **P value** | **Genotype: CC**  **(n=822)** | **Genotype: XT (CT + TT)**  **(n=860)** | **P value** |
| BMI (kg/m^2^) | 24.57±4.66 | 24.43±4.42 | 0. 31^a^ | 24.73±4.64 | 24.32±4.50 | **0.02^a^** |
| WC (cm) | 86.94±11.52 | 87.46±10.99 | 0.96^a^ | 87.15±11.39 | 87.11±11.28 | 0.15^a^ |
| FPG (mg/dl) | 121.26±61.96 | 128.77±63.60 | 0.21^b^ | 119.38±61.37 | 128.42±63.57 | 0.25^b^ |
| HbA1c (%) | 7.09±2.31 | 7.43±2.36 | 0.05^b^ | 7.02±2.28 | 7.40±2.36 | 0.11^b^ |
| Log INS (µIU/ml) | 7.76±1.94 | 7.58±1.86 | 0.52^b^ | 8.12±1.94 | 7.41±1.86 | **0.032^b^** |
| Systolic BP (mmHg) | 123.94±21.23 | 122.86±19.92 | 0.35^b^ | 124.07±20.45 | 123.04±21.05 | 0.27^b^ |
| Diastolic BP (mmHg) | 75.41±11.56 | 75.46±11.83 | 0.94^b^ | 75.52±11.38 | 75.34±11.92 | 0.83^b^ |
| HDL (mg/dl) | 42.43±9.63 | 42.65±9.93 | 0.23^b^ | 42.74±9.59 | 42.30±9.87 | 0.73^b^ |
| LDL (mg/dl) | 119±32.65 | 118.39±34.98 | 0.73^b^ | 118.30±32.49 | 119.24±34.48 | 0.58^b^ |
| VLDL (mg/dl) | 29.85±23.07 | 30.40±22.21 | 0.88^b^ | 29.30±21.66 | 30.77±23.75 | 0.54^b^ |
| TC (mg/dl) | 189.98±39.95 | 189.73±41.48 | 0.86^b^ | 189.35±40.45 | 190.41±40.57 | 0.68^b^ |
| Log TG (mg/dl) | 125.89±1.69 | 128.82±1.69 | 0.41^b^ | 123.02±1.69 | 128.82±1.73 | 0.10^b^ |

Abbreviations: BMI Body mass index; WC waist circumference; FPG Fasting plasmaglucose; HbA1C glycated haemoglobin; INS Fasting plasmainsulin; HDL high density lipoprotein; LDL low density lipoprotein; VLDL very low density lipoprotein; TC Total Cholesterol; TG triglycerides.

Data presented as Mean$\pm$SD

^a^P values are adjusted for age, gender and T2D

^b^P values are adjusted for age, gender, BMI and T2D
