# Supplementary material for: Interaction between TCF7L2 polymorphism and dietary fat intake on high density lipoprotein cholesterol
Source: PLoS One. 2017 Nov 28;12(11):e0188382. doi: 10.1371/journal.pone.0188382 (PMC5705148; doi:10.1371/journal.pone.0188382)
Supplement: S3 Table — (DOCX) [file pone.0188382.s003.docx]

**S3 Table: Association and Interaction between *MC4R* SNP rs17782313 and obesity, T2D and related traits.**

|  | **OR (95% CI)** | **P value** |
| --- | --- | --- |
| Obesity | 0.79 (0.59-1.03) | 0.08^a^ |
| T2D | 1.97 (1.37-2.82) | **0.00022^b^** |
| **Interaction analysis** | **SNP * Carbohydrate (g)** | **SNP * Fibre (g)** |
| Beta coefficients ± Standard error (P_interaction_)**  for interaction on VLDL | -0.19 ± 0.009  (0.045) | -0.24 ± 0.109  (0.027) |
| Beta coefficients ± Standard error (P_interaction_)**  for interaction on TG | -0.10 ± 0.048  (0.042) | -1.20 ± 0.547  (0.028) |

^a^P value is adjusted for age, gender and T2D

^b^P value is adjusted for age, gender and BMI

**P values are adjusted for age, gender, BMI, T2D and Total energy intake

|  | **Genotype: TT**  **(n=488)** | **Genotype: XC**  **(CT + CC)**  **(n=425)** | **P value** |
| --- | --- | --- | --- |
| BMI (kg/m^2^) | 24.65±4.91 | 24.46±4.61 | 0.08^a^ |
| WC (cm) | 86.82±12.85 | 85.87±12.25 | **0.03^a^** |
| FPG (mg/dl) | 109.98±51.19 | 115.45±58.07 | 0.31^b^ |
| HbA1c (%) | 6.59±2.05 | 6.80±2.13 | 0.35^b^ |
| Log INS (µIU/ml) | 7.24±1.86 | 8.12±1.86 | 0.04^b^ |
| Systolic BP (mmHg) | 119.86±19.16 | 120.64±19.29 | 0.84^b^ |
| Diastolic BP (mmHg) | 75.12±11.69 | 74.35±11.49 | 0.37^b^ |
| HDL (mg/dl) | 41.69±9.28 | 42.82±9.65 | 0.16^b^ |
| LDL (mg/dl) | 113.47±33.15 | 114.96±32.22 | 0.88^b^ |
| VLDL (mg/dl) | 28.16±22.33 | 26.91±17.47 | 0.08^b^ |
| TC (mg/dl) | 181.41±39.30 | 184.56±42.27 | 0.67^b^ |
| Log TG (mg/dl) | 116.94±1.73 | 115.34±1.69 | 0.71^b^ |

Abbreviations: BMI Body mass index; WC waist circumference; FPG Fasting plasma glucose; HbA1C glycated haemoglobin; INS Fasting plasma insulin; HDL high density lipoprotein; LDL low density lipoprotein; VLDL very low density lipoprotein; TC Total Cholesterol; TG triglycerides.

Data presented as Mean$\pm$SD

^a^P values are adjusted for age, gender and T2D

^b^P values are adjusted for age, gender, BMI and T2D
