# Supplementary material for: Interaction between TCF7L2 polymorphism and dietary fat intake on high density lipoprotein cholesterol
Source: PLoS One. 2017 Nov 28;12(11):e0188382. doi: 10.1371/journal.pone.0188382 (PMC5705148; doi:10.1371/journal.pone.0188382)
Supplement: S4 Table — (DOCX) [file pone.0188382.s004.docx]

**S4 Table: Interactions between *TCF7L2* SNP rs7903146 and physical activity on VLDL and TG.**

|  | **SNP * physical activity** |
| --- | --- |
| Beta coefficients ± Standard error (P_Interaction_)** for Interaction on VLDL | -4.73 ± 1.890 (0.012) |
| Beta coefficients ± Standard error (P_Interaction_)** for Interaction on TG | -23.24 ± 9.476 (0.014) |

**P values are adjusted for age, gender, BMI and T2D
